# Promoting HPV vaccination at school: a mixed methods study exploring knowledge, beliefs and attitudes of French school staff

**DOI:** 10.1186/s12889-023-15342-2

**Published:** 2023-03-14

**Authors:** Aurélie Bocquier, Marion Branchereau, Aurélie Gauchet, Stéphanie Bonnay, Maïa Simon, Marie Ecollan, Karine Chevreul, Judith E. Mueller, Amandine Gagneux-Brunon, Nathalie Thilly, the PrevHPV Study Group

**Affiliations:** 1 Université de Lorraine, APEMAC, F-54000 Nancy, France; 2 Centre Régional de Coordination Des Dépistages Des Cancers-Pays de La Loire, Angers, France; 3 Université Grenoble Alpes, LIP/PC2S, EA 4145, Grenoble, France; 4 Université Savoie Mont Blanc, LIP/PC2S, Chambéry, France; 5 Département de Médecine Générale, Université de Paris, Faculté de Santé, UFR de Médecine, F-75014 Paris, France; 6 Université de Paris, ECEVE, Paris, France; 7 Assistance Publique-Hôpitaux de Paris, Hôtel Dieu, URC Eco Ile-de-France / Hôpital Robert Debré, Unité d’épidémiologie Clinique, Paris, France; 8 INSERM, ECEVE UMR 1123, Paris, France; 9 Institut Pasteur, Université Paris Cité, Emerging Disease Epidemiology Unit, F-75015 Paris, France; 10 Université Rennes, EHESP, CNRS, Inserm, Arènes - UMR 6051, RSMS (Recherche Sur Les Services Et Management en Santé) - U 1309, Rennes, F-35000 France; 11 Centre International de Recherche en Infectiologie, Team GIMAP, Université Lyon, Université Jean Monnet, Université Claude Bernard Lyon 1, Inserm, U1111, CNRS, UMR530, CIC INSERM 1408 Vaccinologie, CHU de Saint-Etienne, Saint-Etienne, France; 12 Université de Lorraine, CHRU-Nancy, Département Méthodologie, Promotion, Investigation, Nancy, F-54000 France

**Keywords:** Human Papillomavirus, Vaccination, Knowledge, Attitude, Health Education, Middle school professionals, School-based immunization programs, Mixed method study

## Abstract

**Background:**

HPV vaccine coverage in France remained lower than in most other high-income countries. Within the diagnostic phase of the national PrevHPV program, we carried out a mixed methods study among school staff to assess their knowledge, beliefs and attitudes regarding HPV, HPV vaccine and vaccination in general, and regarding schools’ role in promoting HPV vaccination.

**Methods:**

Middle school nurses, teachers and support staff from four French regions participated between January 2020 and May 2021. We combined: (i) quantitative data from self-administered online questionnaires (*n* = 301), analysed using descriptive statistics; and (ii) qualitative data from three focus groups (*n* = 14), thematically analysed.

**Results:**

Less than half of respondents knew that HPV can cause genital warts or oral cancers and only 18% that no antiviral treatment exists. Almost 90% of the respondents knew the existence of the HPV vaccine but some misunderstood why it is recommended before the first sexual relationships and for boys; 56% doubted about its safety, especially because they think there is not enough information on this topic. Schools nurses had greater knowledge than other professionals and claimed that educating pupils about HPV was fully part of their job roles; however, they rarely address this topic due to a lack of knowledge/tools. Professionals (school nurses, teachers and support staff) who participated in the focus groups were unfavourable to offering vaccination at school because of parents’ negative reactions, lack of resources, and perceived uselessness.

**Conclusions:**

These results highlight the need to improve school staff knowledge on HPV. Parents should be involved in intervention promoting HPV vaccination to prevent their potential negative reactions, as feared by school staff. Several barriers should also be addressed before organizing school vaccination programs in France.

**Supplementary Information:**

The online version contains supplementary material available at 10.1186/s12889-023-15342-2.

## Background

Human papillomavirus (HPV) infection is the most common viral infection of the reproductive tract, and a major public health issue [1, 2]. Vaccination is nowadays the most effective strategy to prevent it [2]. As in most high-income countries, HPV vaccination has been included in French vaccination schedules since 2007, initially for girls [3]. In December 2019, the National Authority for Health expanded this recommendation for boys [4]. This recommendation has then been implemented in the French vaccine schedule (and HPV vaccine reimbursed by the Health Insurance for boys) from January 1, 2021 [5]. HPV vaccination is now recommended for all adolescents aged 11–14 years. Despite various efforts by French authorities to promote HPV vaccination these past ten years, vaccine coverage (VC, 24% among 16-year girls in 2018) has remained far lower than in most other high-income countries [3, 6, 7]. In this context, the PrevHPV national research program on the acceptability of HPV vaccination was launched in 2019. It is conducted by a consortium of eight French teams (see list in Additional Table 1) and includes: (i) a “diagnostic” phase, to explore barriers and facilitators to HPV vaccination among different population groups; (ii) a “co-construction” phase, to design a multicomponent intervention to improve HPV VC; and (iii) an “experimental” phase, to evaluate the effectiveness, efficiency and implementation of this intervention.

As part of the PrevHPV program, strengthening the role of schools in promoting HPV vaccination has been identified as a promising way to improve VC [8, 9]. Schools occupy a great part of most adolescents’ life and could play a significant role towards vaccination, from education to vaccine administration [10]. There is currently no nationwide school-based vaccination program in France unlike in most European countries with high HPV VC (e.g., the UK, Scandinavian countries) [3]. Usual pathway to access vaccination in France requires that patients make an appointment with a physician to get the vaccine prescription, go to a community pharmacy to obtain the vaccine, and finally make another appointment for its administration [3]. Regarding education, French pupils learn some basics about immunization and vaccines between grades 7 and 9 as part of the life science curriculum [11]. Pupils should also benefit from three annual sessions (from grades 6 to 9) on sex education. However, these sessions cover many different aspects (biological, psycho-emotional, juridical and social) and all sexual transmitted infections (particularly HIV) [12].

Strengthening the role of school in promoting HPV vaccination require buy-in from all school stakeholders, including teachers, school health staff (generally a part-time nurse in France), administrative and support staff [10]. Together with other barriers (e.g., limited school and vaccination program resources, competing priorities within the school setting, logistical issues), school staff’s poor knowledge about HPV and negative perceptions regarding the HPV vaccine (e.g., concerns about its safety and efficacy) could impair the delivery and effectiveness of school-based HPV vaccination programs [13, 14]. Previous studies conducted abroad (e.g., US, New-Zealand, Italy) have reported a lack of knowledge about HPV among school staff [15–17], including school nurses [18, 19], but French data are lacking to date. Due to the high level of vaccine hesitancy among the French general population [20] as among nurses [21], and controversies that occurred in France around previous vaccination campaigns in schools (e.g., Hepatitis B in the late 1990s [22], influenza A(H1N1) epidemic in 2009 [23]), exploring French school staff knowledge and perceptions about HPV vaccination would be worthwhile.

In this context, as part of the diagnostic phase of the PrevHPV program, we carried out a mixed methods study among school staff to assess their knowledge, beliefs and attitudes regarding HPV, HPV vaccine and vaccination in general, and regarding the role of school in promoting HPV vaccination. The aim was to identify barriers and facilitators, as well as needs of the different school professionals who could be involved in the development and the implementation of interventions promoting HPV vaccination in French middle schools.

## Methods

### Design and setting

We conducted a concurrent mixed methods study [24] combining quantitative data coming from self-administered online questionnaires and qualitative data coming from focus groups. Mixed methods studies are used to assess frequency of outcomes and to have also an in-depth understanding of the underlying processes/experiences [24].

This study was conducted among school staff (nurses, teachers and support staff) from middle schools (pupils typically aged 11–14 years, corresponding to grades 6–9 in the US educational system). We selected schools located in four regions (out of 13 regions in mainland France), hereafter called “study regions”, where the PrevHPV teams were settled and representing a diversity of geographical, demographic and socioeconomic contexts as well as HPV vaccine coverage rates [25]: Ile-de-France (HPV VC among 16-year girls in 2018: 19%), Auvergne-Rhône-Alpes (23%), Grand Est (29%) and Pays de la Loire (30%). The study was planned to be conducted from January to April 2020 but was interrupted due to the Covid-19 pandemic and the schools’ closure in March 2020 in France; it lasted until May 2021. Regarding the quantitative data, we planned to collect 300 questionnaires, a sample size calculated to obtain estimates on knowledge, beliefs and attitudes (expected splits about 70:30 [26]) with a 5% precision and considered feasible in terms of the recruitment. Regarding the qualitative data, we planned to perform three to six focus groups (five to eight participants to each) depending on data saturation [27].

This study was part of study *C19-54* conducted under the responsibility of Inserm. It was granted approval by Evaluation Committee of Inserm, the Institutional Review Board (IRB00003888, IORG0003254, FWA00005831) on 10 December 2019. All study participants gave their informed non opposition to participation, in line with French legal guidelines. This study follows the COREQ (COnsolidated criteria for REporting Qualitative research) and the STROBE (STrengthening the Reporting of OBservational studies in Epidemiology) reporting guidelines (see completed checklists in Additional Tables 2 and 3).

### Participants’ recruitment

First, using data from the Ministry of National Education, we selected middle schools located in the study regions to ensure a balanced distribution of urban/rural areas, public/private schools, and, public schools belonging to a high-priority educational network (high level of social deprivation)/others. Then, we contacted the head of each middle school by email/phone to ask him/her to participate in the study. As we aimed to recruit 30 to 40 middle schools, we first selected 80 schools (expected acceptation rate: 50%) and planned to select additional schools if needed.

Heads of the schools who accepted to participate informed all school staff of the study and sent them the link to the online questionnaire (expected duration: 15 min). Participation of the school staff in the online quantitative survey was voluntary and not compensated.

School staff interested in participating in a focus group were invited to contact the research team by email. The participant information sheet attached to the invitation stated that participants to the focus groups would be offered a 20€ shopping voucher.

### Data collection

#### Quantitative data: self-administered online questionnaire

The questionnaire was administered online using LimeSurvey software. It was designed by the PrevHPV multidisciplinary study group based on the existing literature on determinants of HPV vaccination [26, 28–30] and previous studies among school staff [16, 17]. It included closed-ended questions on (Additional Document 1):

- knowledge about HPV infections: 10 items (*yes*, *no*, *unsure*) and one item on whether cervical cancer is due to a persistent HPV infection (*yes*, *no*, *some cervical cancers only*, *unsure*);
- knowledge about HPV prevention and vaccination: 12 items (*yes*, *no*, *unsure*);
- psychological antecedents of vaccination, assessed using the long-version of the 5C (Confidence, Complacency, Constraints, Calculation and Collective responsibility) scale [31]: 15 items 7-point Likert scale (from 1 = *strongly disagree* to 7 = *strongly agree*);
- personal vaccination status and attitudes towards HPV vaccination: being vaccinated against HPV (*yes*, *no*, *unsure*) and, if *no* or *unsure*, acceptability to receive HPV vaccine if it was possible and recommended for them (5-point Likert scale from 1 = *strongly disagree* to 5 = *strongly agree*); appropriate period to propose HPV vaccination among pupils (*before middle school*, *grade 6*, *grade 7*, *grade 8*, *grade 9*, *never*).

We also collected data on demographic personal and professional characteristics (age, gender, profession) and practices, i.e. the frequency (*always*, *often*, *sometimes*, *never*) they discuss with pupils each of nine different public health topics, including vaccination.

#### Qualitative data: focus groups

The focus groups followed an interview guide (Additional Document 2) composed of open-ended questions exploring participants’ (i) knowledge about HPV and HPV vaccination; (ii) attitudes, preferences and barriers regarding HPV vaccination; and (iii) views regarding the role of school in promoting HPV vaccination. The interview guide was developed through an informal consensus by the study group, based on its expertise in qualitative research regarding attitudes towards HPV vaccination and results from the literature.

We planned to conduct face-to-face focus groups in some selected middle schools but had to propose also one virtual meeting due to the Covid-19 pandemic (expected duration: 1h30). Each focus group was conducted by two members of the study group (MB, M.Soc.S., JB, Ph.D.; and/or CJ, Ph.D.) trained in qualitative research interviews. After an oral consent, all the focus groups were recorded and transcribed.

### Data analysis and interpretation

Descriptive statistics were used to describe characteristics of the participants and their responses to the online questionnaire. Quantitative variables are presented as means and standard deviations and categorical variables as numbers and percentages. Knowledge, beliefs and attitudes are presented overall and by profession which are compared by using Chi2 or exact Fisher tests and ANOVA for respectively categorical and quantitative variables. A *p* value of  < 0.05 for two-sided tests was considered significant. All analyses were performed with SAS V.9.4 (SAS Institute, Cary, North Carolina, USA).

A thematic analysis was used to analyse transcripts of the focus groups. An analysis grid was developed by members of the study group (MB, SB, CJ and AG). Each theme and subtheme were discussed until a consensus was reached. Each focus group was then coded according to this grid.

Finally, this mixed approach used a merging data approach [24] to combine the quantitative data in the form of numeric information with the qualitative data in the form of texts for the main themes of the study.

## Results

### Participants’ characteristics

Three hundred and one participants from 17 different schools completed the online questionnaire (average number of participants per school: 18 ± 26; average duration: 10 ± 6 min). About half of them were teachers and one third school nurses. Most (84%) respondents were women and 49% were older than 45 years (Table 1). To reach this sample size, we contacted a total of 83 middle schools. Heads of 35 (42%) schools accepted to participate and then to forward to their staff the link to access to the online questionnaire (see characteristics in Additional Table 4); in 18/35 schools, no school staff completed the questionnaire. Heads of 31 schools refused (main reasons: too heavy workload, especially in the context of the Covid-19 pandemic), and 17 did not answer.

**Table 1:** Characteristics of the participants to the study

**Respondents to the self-administered online questionnaire (*n* = 301)**			
**Characteristics**		**N**	**%**
***Participants’ school characteristics***			
Location			
Rural		184	61
Urban		113	38
NA		4	1
Status			
Public		281	94
Private		16	5
NA		4	1
High-priority educational network^a^			
Yes		40	14
No		241	86
***Professional characteristics***			
Teachers^b^		143	47
School nurses		105	35
Support staff		53	18
***Personal characteristics***			
Gender			
Women		254	84
Men		47	16
Age (years)			
< 30		18	6
[30–45]		136	45
> 45		147	49
**Participants to the focus groups (*n* = 14)**			
**Participant ID**	**Age (years)**	**Gender**	**Profession**
***Focus group 1 (March 2020, face-to-face***^**c**^***)***			
P1	55	Woman	Teacher
P2	60	Man	Support staff (educational)
P3	53	Woman	Support staff (administrative)
P4	38	Woman	Support staff (administrative)
***Focus group 2 (September 2020, face-to-face***^**c**^***)***			
P5	43	Man	Teacher
P6	54	Man	Support staff (administrative)
P7	45	Woman	Teacher
P8	44	Woman	Support staff (administrative)
P9	41	Woman	Teacher
***Focus group 3 (February 2021, virtual***^**d**^***)***			
P10	40	Woman	School nurse
P11	44	Woman	School nurse
P12	38	Woman	School nurse
P13	62	Woman	School nurse
P14	48	Woman	School nurse

*NA* not available

^a^According to the social deprivation level (for public schools only)

^b^Main disciplines taught: French (21%), mathematics (14%) and life sciences (13%)

^c^Participants worked in the same school

^d^Participants worked in five different schools

Three focus groups (14 professionals) were conducted (duration: 50 min to 2 h). Most participants (11/14) were women; 5 were school nurses, 4 teachers and 5 support staff (Table 1).

### Knowledge, beliefs and attitudes towards HPV infections and vaccination

#### Knowledge about HPV infections

Most (80%) respondents to the questionnaire knew that HPV is a sexually transmitted infection that concerns both female and male (Table 2). Less than half knew that HPV can cause genital warts or oral cancers and are responsible for all cervical cancers. Only 18% knew that there is no antiviral treatment against HPV infections.

**Table 2:** Knowledge about HPV among respondents to the self-administered online questionnaire and by profession (n = 295^a^) – in decreasing order of % of correct answer among all participants

	All participants (%)			Nurses (%)	Teachers (%)	Support staff (%)	*p*^b^
Item	Correct answer	Incorrect answer	Unsure	Correct answer	Correct answer	Correct answer	
HPV is a sexually transmitted virus (T)	83	4	13	98	76	75	< .001
HPV also concerns boys and men (T)	79	7	14	94	71	68	< .001
Different types of HPV exist; only some of them cause cancers (T)	63	3	34	83	52	51	< .001
HPV infection is always symptomatic (F)	62	7	31	87	48	49	< .001
More than half men and women are infected by HPV during their life course (T)	47	7	46	70	34	38	< .001
HPV causes genital warts (T)	47	9	44	74	34	30	< .001
Cervical cancer is due to a persistent HPV infection (T)	38	47	15	37	33	51	0.068
HPV can also cause oral cancers (T)	36	13	51	56	23	34	< .001
Most HPV infections can be eliminated spontaneously by our immune system (T)	28	24	48	40	21	25	0.005
There is no antiviral treatment against HPV infections (T)	18	20	62	25	13	19	0.043

*F* false, *T*  true

^a^Missing data: *n* = 6

^b^Chi2 or exact Fisher test

Participants to the focus groups felt very poorly informed about HPV (Table 3 and Additional Table 5), that they often perceived as an infection concerning mainly females. Participants often cited their own general practitioner (GP) as their main source of information about HPV, but media was also mentioned.

**Table 3:** Selection of the most illustrative verbatim of participants to the focus groups (*n* = 3 focus groups, 14 participants)

Theme	Verbatim
***Knowledge about HPV infections***	
Poorly informed	*“We lack a lot of information and even more on boys but also on girls in general”* (P14, nurse)
A female problem	“*Yes, we need to have figures on the risks for boys who do not get vaccinated, I learned it from my gynaecologist who said that it caused oral cancers in boys, well, it's true that I didn't know*” (P14, nurse)
Sources of information	*“I follow the news very regularly […] that's how I heard about the papillomavirus”* (P5, teacher)
***Knowledge, beliefs and attitudes towards HPV vaccination***	
Vaccination schedule	*“But once he had his first intercourse, it was no longer effective, well, maybe I'm wrong, that's what I understood”* (P4, support staff)
Vaccination for boys	*“And why vaccinate boys, what is the point of vaccinating boys? […] we say at the beginning 10 years ago it was girls, now we say boys […] what has changed now it would be boys?… it's unclear”* (P11, nurse)
Vaccine efficacy / safety	*“Because there are so many papillomaviruses and this vaccine concerns only one kind of papillomavirus so is it useful?”* (P11, nurse) “*I'm afraid that it* [the HPV vaccine] *will lead to something else because we don't have enough hindsight*” (P9, teacher)
***Antecedents of vaccination in general***	
Collective Responsibility	*“Yes it’s a public health problem […] I don't know if this vaccination would really eradicate the virus completely if we were all vaccinated […] but in any case it would greatly reduce the number of cancer risks for both girls and boys”* (P14, nurse)
Confidence	*“We have already seen vaccines that have, heu, caused, heu, multiple sclerosis* [referring to the hepatitis B vaccination]*”* (P3, support staff)
Target population	*“Ha bah papillomavirus equals sexuality in everyone's head euh that's it and inevitably ask a little boy/girl who's entering 6th grade, well, let's think about your sexuality, well, it's taboo for many families, right? I'm not sure if we in the 6th grade can talk about this it's complicated”* (P11, nurse)
***Schools’ role in promoting HPV vaccination***	
Informing/educating pupils	A positive attitude among nurses and some teachers/support staff *"Even as part of our teaching, I think it could be interesting […] Ah I think of the sciences, in particular… yes life sciences […] even an external worker euh I think that it could be interesting "* (P6, support staff) *"It's fully within our job, we're not here for minor medical care essentially we're also here for information and prevention”* (P13, nurse) But some reluctance among teachers/support staff *"Well, the school is not necessarily the place to get information about vaccinations […] I think we already do quite a lot”* (P1, teacher)
Offering access to HPV vaccination	*"I don't think it's the school’s role […] I think it's the role of the family’s health professionals”* (P4, support staff) *"Be careful, the school is not a place of care so euh vaccinate, we are alone that is to say that there is no doctor…”* (P11, nurse) *"I would tend to say that this is not the place and that our country allows us to be vaccinated, I mean we have other places”* (P14, nurse)

#### Knowledge, beliefs and attitudes towards HPV vaccination

Almost 90% of the respondents to the questionnaire knew the existence of the HPV vaccine and 56% knew that it is recommended for heterosexual boys in France (Table 4). Regarding HPV vaccine efficacy and safety, 76% of respondents knew that it protects against HPV-related cancers, but far fewer knew protection against genital warts; 56% were unsure about its safety or thought that it has many side effects. Only 21% were aware that condoms do not protect against HPV (Table 4). Ninety-six percent declared to be unvaccinated against HPV (for 82% because the vaccine did not exist at the time they were 11–14 years old).

**Table 4:** Knowledge about prevention of HPV infections and HPV vaccination among respondents to the self-administered online questionnaire and by profession (*n* = 289^a^) – in decreasing order of % of correct answer among all participants

	All participants (%)			Nurses (%)	Teachers (%)	Support staff (%)	*p*^b^
	Correct answer	Incorrect answer	Unsure	Correct answer	Correct answer	Correct answer	
There is a vaccine against HPV (T)	88	2	10	99	83	79	< .001
Getting vaccinated against HPV prone young girls to have sexual relationships (F)	88	1	11	97	83	85	< .001
Cervical screening remains recommended among vaccinated women (T)	87	1	12	97	83	77	< .001
HPV vaccine protects against virus which cause cancers (T)	76	5	19	93	72	58	< .001
HPV vaccine is effective to prevent precancerous lesions of the cervix (T)	63	4	33	79	55	54	< .001
After the first sexual intercourse, it’s too late to get vaccinated against HPV (F)	57	19	24	67	49	58	0.019
HPV vaccine is recommended for heterosexual boys (T)	56	9	35	81	42	46	< .001
HPV vaccine is responsible for many side effects (F)	43	8	49	69	30	29	< .001
HPV vaccine is recommended for MSM or bisexual boys until 26 years old (T)	33	7	60	51	24	23	< .001
HPV vaccine can help eliminate an HPV infection that already exists (F)	32	13	55	49	21	29	< .001
HPV vaccine protects against genital warts (T)	27	16	57	38	24	15	0.008
Condom protects against HPV infections (F)	21	60	19	24	15	29	0.061

*F*  false, *MSM*  men who have sex with men, *T* true

^a^Missing data: *n* = 12

^b^Chi2 or exact Fisher test

Participants to the focus groups were also aware that a vaccine against HPV exists but had some misunderstandings about its schedule, especially the reason why it is recommended before the first sexual relationships and for boys (Table 3 and Additional Table 5). Some participants reported doubts about vaccine efficacy because it protects only against a few types of HPV; some had heard about side effects in the media or wondered whether there is enough information about vaccine safety.

#### Psychological antecedents and perspectives on vaccination in general

Respondents to the questionnaire showed high confidence in vaccination and had positive attitudes towards collective benefits of vaccination (mean scores > 5, on a scale of 1–7). Barriers to vaccination for themselves (complacency and perceived constraints) were low with mean scores < 2.5 (Additional Table 6).

The importance of vaccination for public health was also reported in focus groups. Some participants mentioned past health controversies that occurred in France around vaccination (e.g., Hepatitis B school vaccination campaign in the late 1990s suspended by the French government after suspected cases of multiple sclerosis [22], management of the A(H1N1) epidemic in 2009 [23]) and wondered about skills of French authorities in that field. Participants perceived a high level of vaccine reluctance among the French general population (Table 3 and Additional Table 5).

#### Differences in knowledge, beliefs and attitudes by profession

The questionnaire results showed greater knowledge about HPV and HPV vaccination among nurses (Tables 2 and 4). Nurses also had a higher level of confidence in vaccination in general (Additional Table 6).

Nurses learned about HPV during their initial training but regretted not receiving any other information as part of their work (even during training about sex education or meetings with other school nurses). They get new information through personal research or the media (Table 3 and Additional Table 5).

### The role of school in promoting HPV vaccination

#### Practices and target population

The questionnaire results showed that vaccination was the public health topic least frequently discussed at school, including by nurses (55% of nurses discussed it often or always with pupils vs 90% for screens’ addiction, diet and sexuality, the most commonly topics discussed, Additional Table 7). For the respondents, grade 8 (age: 13–14 years) was more appropriate to propose HPV vaccination than grade 6 (age: 11–12 years) (Additional Table 8).

Many participants to the focus groups stated it is very complicated to discuss about HPV vaccination with young pupils aged 11 because of the link with sexuality (Table 3 and Additional Table 5).

#### The role of school in informing/educating pupils

In the focus groups, nurses claimed that educating pupils about HPV was fully part of their job roles (Table 3 and Additional Table 5) even if some acknowledged that they never (or only briefly) talk about HPV during sessions about sexuality, mainly because of a lack of knowledge/tools.

Views were more mixed among teachers and support staff. Some felt that it should be the role of school to provide such education and others were more reluctant because they feared parents’ reactions or substantial additional workload. Those supporting the involvement of schools in education about HPV perceived that only nurses, teachers in life sciences or external experts were legitimate to deliver such education.

#### The role of school in offering access to HPV vaccination

There was some agreement between professionals that offering HPV vaccination at school does not fall within the schools’ role (Table 3 and Additional Table 5). Perceived barriers related to parents’ negative reactions, stigmatization of vaccinated/non-vaccinated pupils by their peers, and lack of human and material resources (e.g., no physician in schools). Some participants also argued that vaccination is a personal matter that should involve the GP and that other places are available in France to get vaccinated. Participants acknowledged that vaccination at school already occurred in the past in France (e.g., when they were young for the vaccine against tuberculosis, for hepatitis B in the late 1990s or influenza A(H1N1) in 2009) but were not favorable to it, except in exceptional situations like the current Covid-19 pandemic.

## Discussion

This mixed methods study showed a lack of knowledge towards HPV among staff from French secondary schools. A majority of respondents doubted about HPV vaccine safety and some misunderstood why it is recommended before the first sexual relationships and for boys. Schools nurses had greater knowledge than other professionals and claimed that educating pupils about HPV was fully part of their job roles. There was some agreement between professionals that offering HPV vaccination at school does not fall within the schools’ role. Perceived barriers related to parents’ negative reactions, lack of human and material resources, and perception that other places than schools exist in France to get vaccinated.

### Study’s strengths and limitations

This study adds evidence to the few quantitative studies on school staff’s knowledge, beliefs and attitudes towards HPV vaccination [15–19] and, to the best of our knowledge, is the first French study on this topic. Its mixed methods design allowed us to estimate the prevalence of the main outcomes (e.g., knowledge, beliefs, attitudes) and to explore their sources of information and the barriers they perceived regarding HPV education and vaccination at school. This study has also some limitations. First, the multi-level recruitment process (acceptance by the head of the school then by school staff) probably induced a selection bias. Respondents to the quantitative survey are likely to be more interested in health topics than non-respondents and they may be more favorable to HPV vaccination. Our results may thus overestimate actual French school staff’s knowledge and positive beliefs towards HPV vaccination due to selection bias. However, the proportion of respondents who doubted about vaccine safety in our study (56%) is close to that found among a representative sample of French parents of girls aged 11–19 in 2016 (60%) [26]. Besides, as volunteers to participate in the focus groups had to contact the research team, they were likely to be particularly interested in the HPV topic and to have opinions that cannot be generalized to all school professionals. However, we found some variability in participants’ opinions. Our sample also included mostly women, which is consistent with sex balance in such population groups in France (teachers and school nurses) [32]. Finally, we considered teachers as a one group, while those teaching life sciences probably have better knowledge about HPV and vaccination than the others. However, our sample size prevented us to test this hypothesis.

### Implications for the PrevHPV program and for public health

Consist with findings from previous studies conducted in other countries [15–19], we found a lack of knowledge about HPV and some misunderstanding about HPV vaccination among school staff in France. As better knowledge is associated with more positive attitudes towards HPV vaccine [33], improving school staff access to evidence-based HPV and vaccine information (e.g., via eLearning) should be a prerequisite for educating pupils on this topic. Beyond knowledge, improving school staff’s awareness of their influential position regarding pupils’ and parents’ attitudes is worthwhile [33]. As expected, school nurses had better knowledge about HPV and more positive attitudes towards the role of school in health education than teachers/school staff. However, French school nurses are often responsible for a large area including several schools, and work only part-time in each middle school. Thus, efforts should be made to improve knowledge and attitudes of school staff who have more frequent contacts with pupils. In particular, they should be better informed about the role that school has to play in health education as part of the official “educational health pathway” included in “education for citizenship” [34].

HPV school-based vaccination programs have shown to be effective to achieve high vaccine coverage [3] but school staff in our study had somewhat unfavourable attitudes towards such strategy. Perceived barriers included the lack of human and material resources in school; thus, potential future school vaccination programs would require partnerships between the National Education and external professionals to organize vaccination days on school premises. Barriers also included the perception that vaccination in school is not necessary because other places exist in France to get vaccinated. School staff should be made aware that French vaccination pathway remains complex and that offering HPV vaccine in schools can improve accessibility (e.g., no appointment needed and no need for parents to take time off of work) [35]. Besides, school based programs can increase equity in HPV vaccine uptake [35] in a context of persistent social inequalities towards this vaccination in France [36].

As already reported in Canada [13], fear of parents’ negative reactions was a significant barrier to HPV promotion at school in our study. Parents (especially mothers) play a major role in HPV vaccination decision-making [37] and should be involved in interventions promoting HPV vaccination in schools. Parents could be invited to meetings planned outside of school time by school staff and/or local health professionals to get information about HPV and its vaccine [38].

Finally, our results suggest that school staff (especially nurses) have a high level of confidence in vaccination in general. However, we also found that past controversies around vaccination fueled doubts about vaccines safety and impaired trust towards French authorities. Restoring trust in vaccination remains challenging and “one size fit all intervention” does not exist [39], but French authorities should be aware of this situation and make efforts to address it.

## Conclusions

This mixed methods study highlighted the need to improve school staff knowledge on HPV and its vaccination, and the perceptions of teachers and support staff about the role of schools in educating pupils on this topic in France. It also provides key findings on the barriers that should be addressed before organizing HPV school vaccination programs in France.

## Supplementary Information

**Additional file 1: Additional Table 1**. Teams conducting the PrevHPV program (The PrevHPV Consortium). **Additional Table 2**. COREQ (COnsolidated criteria for REporting Qualitative research) Checklist. **Additional Table 3**. STROBE (STrengthening the Reporting of OBservational studies in Epidemiology) Statement — checklist of items that should be included in reports of observational studies. **Additional Table 4**. Characteristics of the middle schools invited to participate in the study and of those which accepted to participate. **Additional Table 5**. Additional illustrative verbatim of participants to the focus groups. **Additional Table 6**. Psychological antecedents of vaccination (5C scale) among participants to the self-administered online questionnaire and by profession. **Additional Table 7**. Public health topics discussed with pupils by participants to the self-administered online questionnaire and by profession. **Additional Table 8.** Appropriate period to propose HPV vaccination among pupils according to participants to the self-administered online questionnaire and by profession. **Additional Document 1**. Self-administered online questionnaire. **Additional Document 2**. Focus groups’ interview guide. **Additional Document 3**. Names of the PrevHPV Study Group’s members.

## Data Availability

The data that support the findings of this study are available from the French National Institute for Health and Medical Research (Inserm) but restrictions apply to the availability of these data, which were used under license for the current study, and so are not publicly available. Data are however available from the authors upon reasonable request and with permission of the Inserm.
