# Supplementary material for: Promoting HPV vaccination at school: a mixed methods study exploring knowledge, beliefs and attitudes of French school staff
Source: BMC Public Health. 2023 Mar 14;23:486. doi: 10.1186/s12889-023-15342-2 (PMC10011782; doi:10.1186/s12889-023-15342-2)
Supplement: Supplementary file 1 — Additional file 1: Additional Table 1. Teams conducting the PrevHPV program (The PrevHPV Consortium). Additional Table 2. COREQ (COnsolidated criteria for REporting Qualitative research) Checklist. Additional Table 3. STROBE (STrengthening the Reporting of OBservational studies in Epidemiology) Statement — checklist of items that should be included in reports of observational studies. Additional Table 4. Characteristics of the middle schools invited to participate in the study and of those which accepted to participate. Additional Table 5. Additional illustrative verbatim of participants to the focus groups. Additional Table 6. Psychological antecedents of vaccination (5C scale) among participants to the self-administered online questionnaire and by profession. Additional Table 7. Public health topics discussed with pupils by participants to the self-administered online questionnaire and by profession. Additional Table 8. Appropriate period to propose HPV vaccination among pupils according to participants to the self-administered online questionnaire and by profession. Additional Document 1. Self-administered online questionnaire. Additional Document 2. Focus groups’ interview guide. Additional Document 3. Names of the PrevHPV Study Group’s members. [file 12889_2023_15342_MOESM1_ESM.docx]

**Additional Material**

**Additional Table 1** Teams conducting the PrevHPV program (The PrevHPV Consortium)

| **Team n°** | **Contact/scientific leader** | **Field of expertise** |
| --- | --- | --- |
| **1** | EA 4360 APEMAC - Université de Lorraine  9 av. de la Forêt de Haye - BP 20199 - 54505 VANDOEUVRE LES NANCY Cedex  Scientific leader, principal investigator: Pr THILLY Nathalie (email: [n.thilly@chru-nancy.fr](mailto:n.thilly@chru-nancy.fr)) | Epidemiology and Public health |
| **2** | Département de Médecine Générale - Université Paris - 24 rue du Faubourg Saint-Jacques -75679 PARIS Cedex 14  Scientific leader: Pr GILBERG Serge (email: [sergegilberg@gmail.com](mailto:sergegilberg@gmail.com)) | Primary Care |
| **3** | Laboratoire Interuniversitaire de Psychologie - UFR Sciences de l'Homme et de la Société - Université Grenoble Alpes  BP 47 - 38040 GRENOBLE Cedex 9  Scientific leader: Dr GAUCHET Aurélie (email: [aurelie.gauchet@univ-grenoble-alpes.fr](mailto:aurelie.gauchet@univ-grenoble-alpes.fr)) | Health Psychology |
| **4** | CRCDC Pays de la Loire  5 rue des Basses Fouassières - 49000 ANGERS  Scientific leader: Dr LE DUC-BANASZUK Anne-Sophie (email: [as.banaszuk@depistagecancers.fr](mailto:as.banaszuk@depistagecancers.fr)) | Public Health, Cancer prevention |
| **5** | Campus Santé Innovations - Faculté de Médecine Jacques Lisfranc 10 rue de la Marandière - 42270 SAINT-PRIEST-EN-JAREZ  Scientific leader: Dr GAGNEUX-BRUNON Amandine (email: [amandine.gagneux-brunon@chu-st-etienne.fr](mailto:amandine.gagneux-brunon@chu-st-etienne.fr)) | Infection Diseases |
| **6** | INSERM UMR 1123 ECEVE, Université de Paris,75010 PARIS  Scientific leader: Pr CHEVREUL Karine (email: [karine.chevreul@inserm.fr](mailto:karine.chevreul@inserm.fr)) | Health Economics |
| **7** | Institut Pasteur - 25 rue du Dr Roux - 75724 Paris cedex 15  Scientific leader: Dr MUELLER Judith (email: judith.mueller@ehesp.fr) | Epidemiology and Public health |
| **8** | CHRU de Tours - Centre d’investigation Clinique Bretonneau - 37044 Tours cedex 9  Scientific leader: Pr GIRAUDEAU Bruno (email: [bruno.giraudeau@univ-tours.fr](mailto:bruno.giraudeau@univ-tours.fr)) | Biostatistics |

**Additional Table 2** COREQ (COnsolidated criteria for REporting Qualitative research) Checklist

| **Topic** | **Item No.** | **Guide Questions/Description** | **Reported on Page No.** |
| --- | --- | --- | --- |
| **Domain 1: Research team and reflexivity** | | | |
| *Personal Characteristics* | | | |
| Interviewer/facilitator | 1 | Which author/s conducted the interview or focus group? | 8 |
| Credentials | 2 | What were the researcher’s credentials? E.g. PhD, MD | 8 |
| Occupation | 3 | What was their occupation at the time of the study? | 8 |
| Gender | 4 | Was the researcher male or female? | 1, 17 |
| Experience and training | 5 | What experience or training did the researcher have? | 8 |
| *Relationship with participants* | | | |
| Relationship established | 6 | Was a relationship established prior to study commencement? | 6-7 |
| Participant knowledge of the interviewer | 7 | What did the participants know about the researcher? e.g. personal goals, reasons for doing the research | 6 |
| Interviewer characteristics | 8 | What characteristics were reported about the interviewer/facilitator? e.g. Bias, assumptions, reasons and interests in the research topic | N/A |
| **Domain 2: Study design** | | | |
| *Theoretical framework* | | | |
| Methodological orientation and Theory | 9 | What methodological orientation was stated to underpin the study? e.g. grounded theory, discourse analysis, ethnography, phenomenology, content analysis | 8 |
| *Participant selection* | | | |
| Sampling | 10 | How were participants selected? e.g. purposive, convenience, consecutive, snowball | 6-7 |
| Method of approach | 11 | How were participants approached? e.g. face-to-face, telephone, mail, email | 6-7 |
| Sample size | 12 | How many participants were in the study? | 9 |
| Non-participation | 13 | How many people refused to participate or dropped out? Reasons? | N/A |
| *Setting* | | | |
| Setting of data collection | 14 | Where was the data collected? e.g. home, clinic, workplace | 8 |
| Presence of non-participants | 15 | Was anyone else present besides the participants and researchers? | N/A |
| Description of sample | 16 | What are the important characteristics of the sample? e.g. demographic data, date | Table 1 |
| *Data collection* | | | |
| Interview guide | 17 | Were questions, prompts, guides provided by the authors? Was it pilot tested? | 8 and Additional Document 2 |
| Repeat interviews | 18 | Were repeat interviews carried out? If yes, how many? | N/A |
| Audio/visual recording | 19 | Did the research use audio or visual recording to collect the data? | 8 |
| Field notes | 20 | Were field notes made during and/or after the interview or focus group? | N/A |
| Duration | 21 | What was the duration of the interviews or focus group? | 9 |
| Data saturation | 22 | Was data saturation discussed? | 9 |
| Transcripts returned | 23 | Were transcripts returned to participants for comment and/or correction? | N/A |
| **Domain 3: Analysis and findings** | | | |
| *Data analysis* | | | |
| Number of data coders | 24 | How many data coders coded the data? | 9 |
| Description of the coding tree | 25 | Did authors provide a description of the coding tree? | N/A |
| Derivation of themes | 26 | Were themes identified in advance or derived from the data? | 9 |
| Software | 27 | What software, if applicable, was used to manage the data? | N/A |
| Participant checking | 28 | Did participants provide feedback on the findings? | N/A |
| *Reporting* | | | |
| Quotations presented | 29 | Were participant quotations presented to illustrate the themes / findings? Was each quotation identified? e.g. participant number | Table 3 and Additional Table 5 |
| Data and findings consistent | 30 | Was there consistency between the data presented and the findings? | 10-12 |
| Clarity of major themes | 31 | Were major themes clearly presented in the findings? | 10-12 |
| Clarity of minor themes | 32 | Is there a description of diverse cases or discussion of minor themes? | 10-12 |

Developed from: Tong A, Sainsbury P, Craig J. Consolidated criteria for reporting qualitative research (COREQ): a 32-item checklist for interviews and focus groups. International Journal for Quality in Health Care. 2007. Volume 19, Number 6: pp. 349 – 357

**Additional Table 3** STROBE (STrengthening the Reporting of OBservational studies in Epidemiology) Statement — checklist of items that should be included in reports of observational studies

|  | Item No. | Recommendation | Page  No. | | | Relevant text from manuscript |
| --- | --- | --- | --- | --- | --- | --- |
| **Title and abstract** | 1 | (*a*) Indicate the study’s design with a commonly used term in the title or the abstract | 1 | | | “a mixed methods study” |
|  |  | (*b*) Provide in the abstract an informative and balanced summary of what was done and what was found | 3 | | | See Methods and Results section |
| Introduction | | | | | |  |
| Background/rationale | 2 | Explain the scientific background and rationale for the investigation being reported | 4-5 | | | N/A |
| Objectives | 3 | State specific objectives, including any prespecified hypotheses | 5 | | | “to assess their knowledge, beliefs and attitudes regarding HPV, HPV vaccine and vaccination in general, and regarding the role of school in promoting HPV vaccination” |
| Methods | | | | | |  |
| Study design | 4 | Present key elements of study design early in the paper | 5-6 | | | “quantitative data coming from self-administered online questionnaires” |
| Setting | 5 | Describe the setting, locations, and relevant dates, including periods of recruitment, exposure, follow-up, and data collection | 5-6 | | | “The study was planned to be conducted from January to April 2020 but […] lasted until May 2021.” |
| Participants | 6 | (*a*) *Cohort study*—Give the eligibility criteria, and the sources and methods of selection of participants. Describe methods of follow-up  *Case-control study*—Give the eligibility criteria, and the sources and methods of case ascertainment and control selection. Give the rationale for the choice of cases and controls  *Cross-sectional study*—Give the eligibility criteria, and the sources and methods of selection of participants | 6-7 | | | “Heads of the schools who accepted to participate informed all school staff of the study and sent them the link to the online questionnaire” |
|  |  | (*b*) *Cohort study*—For matched studies, give matching criteria and number of exposed and unexposed  *Case-control study*—For matched studies, give matching criteria and the number of controls per case | N/A | | | N/A |
| Variables | 7 | Clearly define all outcomes, exposures, predictors, potential confounders, and effect modifiers. Give diagnostic criteria, if applicable | 7-8 | | | See “Quantitative data: self-administered online questionnaire” section |
| Data sources/ measurement | 8* | For each variable of interest, give sources of data and details of methods of assessment (measurement). Describe comparability of assessment methods if there is more than one group | 7-8 | | | See “Quantitative data: self-administered online questionnaire” section |
| Bias | 9 | Describe any efforts to address potential sources of bias | 6 | | | “we selected middle schools located in the study area to insure a balanced distribution of urban/rural areas, public/private schools…” |
| Study size | 10 | Explain how the study size was arrived at | 6 | | | “We planned to collect 300 questionnaires (a sample size judged appropriate to obtain descriptive statistics on knowledge, beliefs and attitudes with enough precision)” |
| Quantitative variables | 11 | Explain how quantitative variables were handled in the analyses. If applicable, describe which groupings were chosen and why | 8 | | See “Data analysis and interpretation” section | |
| Statistical methods | 12 | (*a*) Describe all statistical methods, including those used to control for confounding | 8 | | See “Data analysis and interpretation” section | |
|  |  | (*b*) Describe any methods used to examine subgroups and interactions | 8 | | See “Data analysis and interpretation” section | |
|  |  | (*c*) Explain how missing data were addressed | Tables 2, 4 | | N/A | |
|  |  | (*d*) *Cohort study*—If applicable, explain how loss to follow-up was addressed  *Case-control study*—If applicable, explain how matching of cases and controls was addressed  *Cross-sectional study*—If applicable, describe analytical methods taking account of sampling strategy | N/A | | N/A | |
|  |  | (*e*) Describe any sensitivity analyses | N/A | | N/A | |
| Results | | | | | | |
| Participants | 13* | (a) Report numbers of individuals at each stage of study—eg numbers potentially eligible, examined for eligibility, confirmed eligible, included in the study, completing follow-up, and analysed | 9 | | See Additional Table 4 | |
|  |  | (b) Give reasons for non-participation at each stage | 9 | | “31 refused (main reasons: too heavy workload, especially in the context of the Covid-19 pandemic)” | |
|  |  | (c) Consider use of a flow diagram | N/A | | N/A | |
| Descriptive data | 14* | (a) Give characteristics of study participants (eg demographic, clinical, social) and information on exposures and potential confounders | 9 | | See “Participants’ characteristics” section | |
|  |  | (b) Indicate number of participants with missing data for each variable of interest | Tables 2, 4 | | N/A | |
|  |  | (c) *Cohort study*—Summarise follow-up time (eg, average and total amount) | N/A | | N/A | |
| Outcome data | 15* | *Cohort study*—Report numbers of outcome events or summary measures over time | N/A | | N/A | |
|  |  | *Case-control study—*Report numbers in each exposure category, or summary measures of exposure | N/A | | N/A | |
|  |  | *Cross-sectional study—*Report numbers of outcome events or summary measures | N/A | | N/A | |
| Main results | 16 | (*a*) Give unadjusted estimates and, if applicable, confounder-adjusted estimates and their precision (eg, 95% confidence interval). Make clear which confounders were adjusted for and why they were included | N/A | | N/A | |
|  |  | (*b*) Report category boundaries when continuous variables were categorized | N/A | | N/A | |
|  |  | (*c*) If relevant, consider translating estimates of relative risk into absolute risk for a meaningful time period | N/A | | N/A | |
| Other analyses | 17 | Report other analyses done—eg analyses of subgroups and interactions, and sensitivity analyses | 11 | See “Differences in knowledge, beliefs and attitudes by profession” section | | |
| Discussion | | | | | | |
| Key results | 18 | Summarise key results with reference to study objectives | 13 | “This mixed methods study showed…” | | |
| Limitations | 19 | Discuss limitations of the study, taking into account sources of potential bias or imprecision. Discuss both direction and magnitude of any potential bias | 13 | See “Study’s strengths and limitations” section | | |
| Interpretation | 20 | Give a cautious overall interpretation of results considering objectives, limitations, multiplicity of analyses, results from similar studies, and other relevant evidence | 14-15 | See “Implications for the PrevHPV program and for public health” section | | |
| Generalisability | 21 | Discuss the generalisability (external validity) of the study results | 13 | See “Study’s strengths and limitations” section | | |
| Other information |  | | | | | |
| Funding | 22 | Give the source of funding and the role of the funders for the present study and, if applicable, for the original study on which the present article is based | 16 | See “Funding” section | | |

*Give information separately for cases and controls in case-control studies and, if applicable, for exposed and unexposed groups in cohort and cross-sectional studies.

**Additional Table 4** Characteristics of the middle schools invited to participate in the study and of those which accepted to participate

| **Characteristics** | **Schools contacted to participate (n = 83)**  **N (%)** | **Schools which accepted to participate to the study (n = 35)**  **N (%)** | **Schools with at least one staff member who completed the online questionnaire (n = 17)**  **N (%)** |
| --- | --- | --- | --- |
| Location |  |  |  |
| Rural | 17 (20) | 10 (29) | 6 (35) |
| Urban | 66 (80) | 25 (71) | 10 (59) |
| NA | / | / | 1 (6) |
| Status |  |  |  |
| Public | 69 (83) | 30 (86) | 13 (76) |
| Private | 14 (17) | 5 (14) | 3 (18) |
| NA | / | / | 1 (6) |
| High-priority educational network^a^ |  |  |  |
| Yes | 19 (28) | 6 (20) | 3 (23) |
| No | 50 (72) | 24 (80) | 10 (77) |
| Region |  |  |  |
| Ile de France | 20 (24) | 8 (23) | 1 (6) |
| Auvergne-Rhône-Alpes | 33 (40) | 13 (37) | 7 (41) |
| Grand Est | 13 (16) | 7 (20) | 3 (18) |
| Pays de Loire | 17 (20) | 7 (20) | 5 (29) |
| NA | / | / | 1 (6) |

NA = not available.

^a^ According to the social deprivation level (for public schools only).

**Additional Table 5** Additional illustrative verbatim of participants to the focus groups (n = 3 focus groups, 14 participants)

| **Theme** | **Verbatim** |
| --- | --- |
| ***Knowledge about HPV infections*** | |
| Poorly informed | *“I had the impression […] only a few people were affected by this kind of diseases, I realize that it’s perhaps not the case, I don’t know”* (P2, support staff) |
| A female problem | “*It's an infection mostly in girls not in boys for me*” (P3, support staff) |
| Sources of information | “*During a consultation with our general practitioner, she clearly told me and my daughter about it, she explained to us what this infection was*” (P4, support staff)  *“[…] even in our training sessions on sex education, they don't talk to us about it, they already talk to us about AIDS, but [...] HPV they don't talk about it”* (P11, nurse) |
| ***Knowledge, beliefs and attitudes towards HPV vaccination*** | |
| Vaccination schedule | *“But once he had his first intercourse, it was no longer effective, well, maybe I'm wrong, that's what I understood”* (P4, support staff) |
| Vaccination for boys | *“I was surprised some time ago after a visit to my general practitioner for my son to learn that boys could also be vaccinated”* (P1, teacher) |
| Vaccine efficacy / safety | *“Because there are so many papillomaviruses and this vaccine concerns only one kind of papillomavirus so is it useful?”* (P11, nurse)  *“I thought that at one time the vaccine has even been called into question […] we were not sure that the vaccine was not finally harmful”* (P2, support staff)  “*I'm afraid that it* [the HPV vaccine] *will lead to something else because we don't have enough hindsight*” (P9, teacher) |
| ***Antecedents of vaccination in general*** | |
| Collective Responsibili-ty | *“Yes it’s a public health problem […] I don't know if this vaccination would really eradicate the virus completely if we were all vaccinated […] but in any case it would greatly reduce the number of cancer risks for both girls and boys”* (P14, nurse) |
| Confidence | *"The famous* [H1N1] *flu where finally there were heu I don't know how many tens of millions of vaccine stocks that were thrown away [...] to show that our leaders are not necessarily very...”* (P2, support staff)  *“I have the impression that there are more and more of them and honestly, it scares me because I have also had people against vaccines in the teachers"* (P14, nurse) |
| Target population | *"And it’s young… she* [her 11 year old daughter] *was embarrassed when I told her about sexual intercourse and she looked at me with big eyes”* (P4, support staff) |
| ***Schools’ role in promoting HPV vaccination*** | |
| Informing/educating pupils | A positive attitude among nurses and some teachers/support staff  *“I'm thinking of middle school as well* [as a way to educate pupils about HPV]. *Because they are quite sensitive to what they are told in schools”* (P9, teacher)  *"If I had more knowledge about HPV, if I could talk about it more convincingly, I would be able to add it as an unavoidable topic to my sex education activities* (P14, nurse)  But some reluctance among teachers/support staff  *“I think, perhaps, that parents would be surprised to have public health information coming into the school”* (P4, support staff)  *“We’ve to be very careful not to enter the parental sphere”* (P2, support staff) |
| Offering access to HPV vaccination | *“Well, then I think it's the parents who are going to, euh, step up to the plate”* (P5 and P7, teachers)  *“if it* [the vaccination] *is done at school, I think that it should also be a little, not anonymous, but not everyone should see who is going to be vaccinated and who is not, so that it is not [...] Yeah, stigmatizing”* (P9, teacher)  *"if we're told we have to vaccinate for the Covid, well, because it's supervised, because that's how it is, because there's a doctor not far away, okay, we've already done it for the H1N1 flu, but to be part of a vaccination policy, to vaccinate, well, I don't know if that's really our role in fact"* (P11, nurse) |

**Additional Table 6** Psychological antecedents of vaccination (5C scale^a^) among participants to the self-administered online questionnaire and by profession (n = 301)

| **Item** | **Mean (standard deviation)** | | | | |
| --- | --- | --- | --- | --- | --- |
|  | **All participants** | **Nurses** | **Teachers** | **Support staff** | ***p*^b^** |
| ***Confidence*** |  |  |  |  |  |
| I am completely confident that vaccines are safe | 5.5 (1.6) | 5.9 (1.2) | 5.5 (1.6) | 4.8 (2.0) | <.001 |
| Vaccinations are effective | 6.0 (1.3) | 6.2 (1.0) | 6.0 (1.3) | 5.3 (1.7) | <.001 |
| Regarding vaccines, I am confident that public authorities decide in the best interest of the community | 5.4 (1.6) | 5.6 (1.5) | 5.4 (1.6) | 4.9 (1.8) | 0.020 |
| ***Complacency*** |  |  |  |  |  |
| Vaccination is unnecessary because vaccine-preventable diseases are not common anymore | 1.9 (1.3) | 1.8 (1.2) | 1.8 (1.2) | 2.3 (1.6) | 0.089 |
| My immune system is so strong; it also protects me against diseases | 2.2 (1.5) | 2.1 (1.4) | 2.0 (1.4) | 2.5 (1.8) | 0.097 |
| Vaccine-preventable diseases are not so severe that I should be vaccinated | 1.9 (1.3) | 1.7 (1.1) | 1.9 (1.3) | 2.5 (1.6) | 0.001 |
| ***Constraints*** |  |  |  |  |  |
| Everyday stress prevents me from being vaccinated | 1.9 (1.3) | 1.8 (1.2) | 1.8 (1.2) | 2.2 (1.6) | 0.167 |
| For me, it is inconvenient to be vaccinated | 2.1 (1.5) | 2.0 (1.5) | 2.2 (1.4) | 2.3 (1.5) | 0.290 |
| Visiting the doctor makes me feel uncomfortable; this keeps me from being vaccinated | 1.6 (1.1) | 1.6 (0.8) | 1.6 (1.1) | 1.8 (1.4) | 0.262 |
| ***Calculation*** |  |  |  |  |  |
| When I think about being vaccinated, I weigh its benefits and risks to make the best decision possible | 5.5 (1.7) | 5.7 (1.6) | 5.3 (1.7) | 5.4 (1.6) | 0.158 |
| For each and every vaccination, I closely consider whether it is useful for me | 5.4 (1.6) | 5.7 (1.5) | 5.3 (1.6) | 5.1 (1.8) | 0.026 |
| It is important for me to fully understand the topic of vaccination before I get vaccinated | 5.9 (1.3) | 6.1 (1.2) | 5.7 (1.5) | 5.8 (1.2) | 0.059 |
| ***Collective responsibility*** |  |  |  |  |  |
| When everyone else is vaccinated, I don’t have to be vaccinated, too (R) | 6.3 (1.2) | 6.3 (1.0) | 6.3 (1.2) | 6.2 (1.4) | 0.760 |
| I get vaccinated because I can also protect people with a weaker immune system | 5.4 (1.8) | 5.3 (1.7) | 5.5 (1.7) | 5.1 (2.1) | 0.300 |
| Vaccination is a collective action to prevent the spread of diseases | 6.3 (1.2) | 6.4 (1.0) | 6.3 (1.3) | 6.1 (1.4) | 0.273 |

^a^ Instruction for the 5C scale: “Please evaluate how much you disagree or agree with the following statements.” (1=*strongly disagree*, 2=*moderately disagree*, 3=*slightly disagree*, 4=*neutral*, 5=*slightly agree*, 6=*moderately agree*, 7=*strongly agree*). Item with (R) is reverse-coded: as all items are scored in such a way that a higher score indicates a higher degree of the “C” assessed, the item “When everyone else is vaccinated, I don’t have to be vaccinated, too” of the “Collective responsibility” subscale is reverse-coded.

^b^ ANOVA.

**Additional Table 7** Public health topics discussed with pupils by participants to the self-administered online questionnaire and by profession (n = 301) - % of “often” or “always”

|  | **All participants (%)** | **Nurses (%)** | | **Teachers (%)** | **Support staff (%)** | | ***p***^a^ |
| --- | --- | --- | --- | --- | --- | --- | --- |
| Screens | 57 | 92 | 34 | | | 53 | <.001 |
| Diet | 55 | 90 | 33 | | | 47 | <.001 |
| Health risks in general | 47 | 82 | 23 | | | 42 | <.001 |
| Physical activity | 45 | 74 | 30 | | | 28 | <.001 |
| Sexuality | 45 | 90 | 19 | | | 26 | <.001 |
| Tobacco consumption | 42 | 70 | 22 | | | 36 | <.001 |
| Drug consumption | 39 | 64 | 20 | | | 40 | <.001 |
| Alcohol consumption | 36 | 60 | 17 | | | 36 | <.001 |
| Vaccination | 28 | 55 | 13 | | | 13 | <.001 |

^a^ Chi2 or exact Fisher test.

**Additional Table 8** Appropriate period to propose HPV vaccination among pupils according to participants to the self-administered online questionnaire and by profession (n = 283) - % of “yes”

|  | **All participants (%)** | **Nurses (%)** | | **Teachers (%)** | **Support staff (%)** | | ***p***^a^ |
| --- | --- | --- | --- | --- | --- | --- | --- |
| Before middle school | 6 | 7 | 4 | | | 6 | 0.038 |
| Grade 6 | 26 | 39 | 20 | | | 18 | 0.001 |
| Grade 7 | 39 | 45 | 36 | | | 31 | 0.192 |
| Grade 8 | 52 | 46 | 61 | | | 39 | 0.013 |
| Grade 9 | 37 | 33 | 39 | | | 39 | 0.636 |
| Never | 4 | 4 | 3 | | | 4 | 0.076 |

^a^ Chi2 or exact Fisher test.

**Additional Document 1** Self-administered online questionnaire

1. How old are you?
   - <30
   - 30-45
   - >45
2. You are:
   - A woman
   - A man
3. When did you finish your studies?
   - Before 1990
   - Between 1990 and 2000
   - Between 2000 and 2010
   - Between 2010 and now
4. What is your profession?
   - Teacher
     - If you are a teacher, which discipline(s) do you teach?: French / Mathematics / History-Geography / Foreign language / Life sciences / Physics-Chemistry / Technology / Music / Art / Physical activity
   - Nurse
   - Physician
   - Other
5. Do you usually address the following prevention topics with pupils?

|  | Always | Often | Sometimes | Never |
| --- | --- | --- | --- | --- |
| 1. Tobacco consumption |  |  |  |  |
| 1. Diet |  |  |  |  |
| 1. Physical activity |  |  |  |  |
| 1. Alcohol consumption |  |  |  |  |
| 1. Vaccination |  |  |  |  |
| 1. Sexuality |  |  |  |  |
| 1. Drugs consumption |  |  |  |  |
| 1. Screens |  |  |  |  |
| 1. At-risk behaviors in general |  |  |  |  |

*Vaccination and you*

1. Please evaluate how much you disagree or agree with the following statements

|  | *Strongly disagree* | *Mode-rately dis-agree* | *Slightly dis-agree* | *Neutral* | *Slightly agree* | *Mode-rately agree* | *Stron-gly agree* |
| --- | --- | --- | --- | --- | --- | --- | --- |
|  |  |  |  |  |  |  |  |
| 1. I am completely confident that vaccines are safe |  |  |  |  |  |  |  |
| 1. Vaccinations are effective |  |  |  |  |  |  |  |
| 1. Regarding vaccines, I am confident that public authorities decide in the best interest of the community |  |  |  |  |  |  |  |
| 1. Vaccination is unnecessary because vaccine-preventable diseases are not common anymore |  |  |  |  |  |  |  |
| 1. My immune system is so strong; it also protects me against diseases |  |  |  |  |  |  |  |
| 1. Vaccine-preventable diseases are not so severe that I should be vaccinated |  |  |  |  |  |  |  |
| 1. Everyday stress prevents me from being vaccinated |  |  |  |  |  |  |  |
| 1. For me, it is inconvenient to be vaccinated |  |  |  |  |  |  |  |
| 1. Visiting the doctor makes me feel uncomfortable; this keeps me from being vaccinated |  |  |  |  |  |  |  |
| 1. When I think about being vaccinated, I weigh its benefits and risks to make the best decision possible |  |  |  |  |  |  |  |
| 1. For each and every vaccination, I closely consider whether it is useful for me |  |  |  |  |  |  |  |
| 1. It is important for me to fully understand the topic of vaccination before I get vaccinated |  |  |  |  |  |  |  |
| 1. When everyone else is vaccinated, I don’t have to be vaccinated, too (R) |  |  |  |  |  |  |  |
| 1. I get vaccinated because I can also protect people with a weaker immune system |  |  |  |  |  |  |  |
| 1. Vaccination is a collective action to prevent the spread of diseases |  |  |  |  |  |  |  |

*HPV infection and vaccination – knowledge*

1. For each of the following statement, please choose one answer:

|  | Yes | No | Unsure |
| --- | --- | --- | --- |
| 1. HPV is a sexually transmitted virus |  |  |  |
| 1. HPV also concerns boys and men |  |  |  |
| 1. More than half men and women are infected by HPV during their life course |  |  |  |
| 1. HPV infection is always symptomatic |  |  |  |
| 1. HPV causes genital warts |  |  |  |
| 1. Different types of HPV exist; only some of them cause cancers |  |  |  |
| 1. HPV can also cause oral cancers |  |  |  |
| 1. Most HPV infections can be eliminated spontaneously by our immune system |  |  |  |
| 1. There is no antiviral treatment against HPV infections |  |  |  |
| 1. There is a vaccine against HPV infections |  |  |  |

1. Cervical cancer is due to a persistent HPV infection
   - Yes
   - No
   - Partially (some cervical cancers only)
   - Unsure
2. For each of the following statement, please choose one answer:

|  | Yes | No | Unsure |
| --- | --- | --- | --- |
| 1. HPV vaccine protects against virus which cause cancers |  |  |  |
| 1. HPV vaccine protects against genital warts |  |  |  |
| 1. HPV vaccine can help eliminate an HPV infection that already exists |  |  |  |
| 1. Cervical screening remains recommended among vaccinated women |  |  |  |
| 1. HPV vaccine is recommended for girls and boys aged 11-14 years with a possible catch-up until 19 years |  |  |  |
| 1. HPV vaccine is recommended for MSM or bisexual boys until 26 years old |  |  |  |
| 1. Condom protects against HPV infections |  |  |  |
| 1. HPV vaccine is responsible for many side effects |  |  |  |
| 1. After the first sexual intercourse, it’s too late to get vaccinated against HPV |  |  |  |
| 1. HPV vaccine is effective to prevent precancerous lesions of the cervix |  |  |  |
| 1. HPV vaccine is recommended for heterosexual boys |  |  |  |
| 1. Getting vaccinated against HPV prone young girls to have sexual relationships |  |  |  |

*HPV infection and vaccination – Attitude*

1. Do you have a vaccination booklet and /or a health booklet?
   - Yes
   - No
   - Unsure
2. Have you heard about the electronic vaccination booklet?
   - Yes
     - If yes, do you have an electronic vaccination booklet? Yes / No / Unsure.
   - No
   - Unsure
3. Are you vaccinated against HPV?
   - Yes
   - No
   - Unsure
     - If you are not vaccinated, please indicate why (several choices possible): The vaccine was not recommended for me / By personal choice / I did have my parents’ agreement / For religious reasons / Because of a lack of time / I did not know that a vaccine exist / I followed my doctor’s recommendation / I preferred to wait / I need additional information / I did not want this vaccine / I was not at risk regarding HPV infection / I was afraid of shots / I was afraid of potential side effects / This vaccine was not effective / The vaccine did not exist at that time
     - Please indicate why you have been vaccinated against HPV or whether you would have been vaccinated if it has been recommended for you (several choices possible): By personal choice / Because of my parents’ recommendation / Because of my doctor’s recommendation / I felt at risk towards HPV / I wanted to avoid a cancer / I did not want to transmit HPV to my sexual partner / I did not want to get vaccinated
4. In which class do you think it is appropriate to propose HPV vaccination?
   - In 6ème
   - In 5ème
   - In 4ème
   - In 3ème
   - Before middle school
   - Never
5. Please evaluate how much you disagree or agree with the following statements

|  | *Strongly disagree* | *Disagree* | *Neutral* | *Agree* | *Strongly agree* |
| --- | --- | --- | --- | --- | --- |
| 1. I recommend to my family and friends targeted by the recommendations to get vaccinated against HPV |  |  |  |  |  |
| 1. If it was possible and recommended for me, I would like to get vaccinated against HPV |  |  |  |  |  |
| 1. Before getting the HPV vaccine or encouraging a relative to get the vaccine, I ask my doctor for advice |  |  |  |  |  |
| 1. Before getting the HPV vaccine or encouraging a relative to get the vaccine, I look for information on the internet |  |  |  |  |  |

**Additional Document 2** Focus groups’ interview guide

| **Theme** | **Questions** |
| --- | --- |
| **Knowledge/beliefs** | **Starting question: What do you know about HPV and its vaccine?**   - *Knowledge about the new recommendations:* **Who should be offered vaccination?** - *Sources of information:* **How and when have heard about the HPV vaccine?** - *Understanding of the vaccine*: **What questions do you still have about this vaccine?** |
| **Attitudes, preferences and barriers to HPV vaccination** | **Starting question: What do you think of this vaccine in terms of public health?**   - *Universal vaccination:* **Do you think that HPV vaccination for boys is warranted?** - *Acceptability of the vaccine***: Why do you think adolescents should be vaccinated?** - *Barriers:* **What are the barriers to HPV vaccination?** |
| **Decision making process** | **Starting question: In your experience, are pupils involved in the decision to get vaccinated?**   - To what extent are pupils seeking information about HPV vaccination? - In your opinion, do parents take into account their child's point of view? |
| **School’s mobilization and participation** | **Starting question: What would you suggest to improve HPV vaccination coverage?**   - *The role of school***: How can schools help promote HPV vaccination?**   - What are your experiences in that field?   - Do you think the school can have an informational/educational role?   - What do you think about vaccination at school? - *Anti-Vax* : **How do you deal with the Anti-vax position?**   **Do you have anything to add?** |

**Additional Document 3** Names of the PrevHPV Study Group’s members

The PrevHPV Study group includes the authors of the present manuscript and: For the team 1: Nelly AGRINIER^1^, Estelle FALL^1^, Céline PULCINI^1,13^; for the team 2: Sébastien BRUEL^5^, Serge GILBERG^5^, Josselin LE BEL^5^, Henri PARTOUCHE^5^, Juliette PINOT^5^, Louise ROSSIGNOL^5^, Arthur TRON^5^, Minghui ZUO^5^; for the team 3: Julie BROS^3,4^, Olivier EPAULARD^14^, Catherine JUNEAU^3,4^, Gaëlle VAREILLES^15^; for the team 4: Anne-Sophie LE DUC-BANASZUK^2^; for the team 5: Elisabeth BOTELHO-NEVERS^11^, Florian JEANLEBOEUF^11^, Julie KALECINSKI^16,17^, Christine LASSET^18,19,20^, Laetitia MARIE DIT ASSE^19^, Mabrouk NEKAA^21^; for the team 6: Morgane MICHEL^6,7,8^; for the team 7: Anne-Sophie BARRET^22^, Isabelle BONMARIN^22^, Sandra CHYDERIOTIS^9^, Daniel LEVY-BRUHL^22^, Jocelyn RAUDE^10^, Jonathan SICSIC^23^; for the team 8: Bruno GIRAUDEAU^24^; Clémence CASTAGNET^25,26^ and Mélanie SIMONY^26^.

^13^ Université de Lorraine, CHRU-Nancy, Service des maladies infectieuses et tropicales, F-54000 Nancy, France.

^14^ CHUGA - service infectiologie, La Tronche, France.

^15^ Centre départemental de santé de l’Isère, Grenoble, France.

^16^ Université Claude Bernard Lyon 1, Research on Healthcare Performance (RESHAPE), INSERM, U1290, Lyon, France.

^17^ Institut de Cancérologie Lucien Neuwirth, Saint-Priest-en-Jarez, France.

^18^ Université Lyon 1, Faculté de Médecine et Maïeutique Charles Mérieux Lyon Sud, Oullins, France.

^19^ Centre Léon Bérard, Département Prévention Santé Publique - DPSP, Lyon, France.

^20^ UMR CNRS 5558 Laboratoire de Biométrie et Biologie Evolutive, Villeurbanne, France.

^21^ French Academic Directorate of the National Education Services of the Loire Department, Univ. Lyon, Université Saint-Etienne, HESPER EA 7425, F-69000 Lyon, France.

^22^ Santé publique France, Saint-Maurice, France.

^23^ LIRAES (EA 4470), University of Paris, Paris, France.

^24^ Université de Tours, Université de Nantes, INSERM, SPHERE U1246, Tours, France ; INSERM CIC1415, CHRU de Tours, Tours, France.

^25^ French National Institute for Health and Medical Research (Inserm), Paris, France.

^26^ French Institute for Public Health Research (IReSP), Paris, France.
